# Carbonic anhydrase 9 is associated with chemosensitivity and prognosis in breast cancer patients treated with taxane and anthracycline

**DOI:** 10.1186/1471-2407-14-400

**Published:** 2014-06-04

**Authors:** Naoki Aomatsu, Masakazu Yashiro, Shinichiro Kashiwagi, Hidemi Kawajiri, Tsutomu Takashima, Masahiko Ohsawa, Kenichi Wakasa, Kosei Hirakawa

**Affiliations:** 1 Department of Surgical Oncology, Osaka City University Graduate School of Medicine, Osaka, Japan; 2 Oncology Institute of Geriatrics and Medical Science, Osaka City University Graduate School of Medicine, 1-4-3 Asahi-machi, Abeno-ku, Osaka 545-8585, Japan; 3 Department of Diagnostic Pathology, Osaka City University Graduate School of Medicine, 1-4-3 Asahi-machi, Abeno-ku, Osaka, Japan

**Keywords:** Breast cancer, Carbonic anhydrase 9, Neoadjuvant chemotherapy, Predictive marker, Chemosensitivity

## Abstract

**Background:**

Neoadjuvant chemotherapy (NAC) is one of the standard care regimens for patients with resectable early-stage breast cancer. It would be advantageous to determine the chemosensitivity of tumors before initiating NAC. One of the parameters potentially compromising such chemosensitivity would be a hypoxic microenvironment of cancer cells. The aim of this study was thus to clarify the correlation between expression of the hypoxic marker carbonic anhydrase-9 (CA9) and chemosensitivity to NAC as well as prognosis of breast cancer patients.

**Methods:**

A total of 102 patients with resectable early-stage breast cancer was treated with NAC consisting of FEC (5-fluorouracil, epirubicin, and cyclophosphamide) followed by weekly paclitaxel before surgery. Core needle biopsy (CNB) specimens and resected tumors were obtained from all patients before and after NAC, respectively. Chemosensitivity to NAC and the prognostic potential of CA9 expression were evaluated by immunohistochemistry.

**Results:**

CA9 positivity was detected in the CNB specimens from 47 (46%) of 102 patients. The CA9 expression in CNB specimens was significantly correlated with pathological response, lymph node metastasis, and lymph-vascular invasion. Multivariate analysis revealed that the CA9 expression in CNB specimens was an independent predictive factor for pathological response. The Kaplan-Meier survival curve revealed a significant negative correlation (p = 0.013) between the disease-free survival (DFS) and the CA 9 expression in resected tissues after NAC. Multivariate regression analyses indicated that the CA9 expression in resected tissues was an independent prognostic factor for DFS.

**Conclusions:**

CA9 expression in CNB specimens is a useful marker for predicting chemosensitivity, and CA9 expression in resected tissue is prognostic of DFS in patients with resectable early-stage breast cancer treated by sequential FEC and weekly paclitaxel prior to resection.

## Background

Neoadjuvant chemotherapy (NAC) increases the rate of breast-conserving surgery and decreases the risk of postoperative recurrence as effectively as adjuvant chemotherapy; thus, it might be considered to enhance survival [1,2]. For this reason, NAC has been one of the standard care regimens for patients with various types of carcinomas, including resectable early-stage breast cancer [3]. The optimal regimen for NAC in breast cancer involves a sequential or concomitant anthracycline-containing regimen and taxane [4,5]. The aim of NAC for breast cancer is to reduce the size of the primary tumor, thereby increasing the likelihood of breast conservation [6], and might allow evaluation of the therapeutic effects that facilitate the strategies of post-operative chemotherapy [7]. Recent studies have demonstrated that the response status after NAC is correlated with improved disease-free survival (DFS) and overall survival (OS) in breast tumors [5,8]. NAC for breast cancer has a pathologic complete response (pCR) rate of approximately 30% [6,9,10] and a clinical complete response (cCR) rate of approximately 60% [10]. In contrast, NAC is ineffective in approximately half of all patients, and many experience toxicity. Therefore, it would be advantageous to identify patients with chemosensitive tumors before initiating NAC, to avoid potential therapy-related complications and an inappropriate delay of surgical treatment.

NAC has numerous advantages, including the provision of pathological response data that can be used as a surrogate marker for long-term clinical outcomes [11,12]. Also, the assessment of responsiveness to NAC allows the evaluation of potential predictive molecular markers for chemosensitivity. Several biological markers, including the estrogen receptor (ER), progesterone receptor (PgR), HER2, Ki-67, p21, p53, Bcl, multi-drug-resistant P-glycoprotein, and topoisomerase 2A, have recently been investigated; however, there exists no clear correlation between the expression of these markers and chemosensitivity after sequential taxane- and anthracycline-based chemotherapies [13-17].

Carbonic anhydrase 9 (CA9) is a cell surface enzyme that catalyzes the reversible hydration of carbon dioxide to bicarbonate and a proton [18] and maintains pericellular pH homeostasis [19,20]. CA 9 is overexpressed in response to tumor hypoxia in many common tumor types [[21-24] and plays a critical role in hypoxia-associated tumor acidosis [[25-27]. Hypoxia-inducible factor-1 α (HIF-1 α) binds to the hypoxia-responsive element present in the promoter regions of CA9 and up-regulates CA9 expression [24,28]. Hypoxia plays an important role in tumor progression and chemoresistance in various types of cancer [29-32]. CA9 has been implicated in the regulation of the micro-environmental pH in tumor hypoxia. In this retrospective study, we examined the correlation between CA9 expression and chemosensitivity to NAC in breast cancer as well as the prognosis of patients.

## Methods

### Patients

A total of 102 patients with resectable early-stage breast cancer, which was considered to be stage IIA (T2 N0 M0), IIB (T2 N1 M0 or T3 N0 M0), or IIIA (T3 N1 M0), were treated with NAC from 2004 to 2009. Breast cancers were confirmed histopathologically by core needle biopsy (CNB) and were staged by computed tomography and bone scan. The clinicopathologic features of the 102 breast cancers are shown in Additional file 1: Table S1. The clinical stage was based on the TNM Classification of Malignant Tumors, 6th Edition [33]. No patients had evidence of distant metastasis at the time of surgery. All of the cases received neoadjuvant chemotherapy consisting of 4[cycles of 5-fluorouracil (5FU) 500 mg/m^2^, epirubicin 75 or 100 mg/m^2^, and cyclophosphamide 500 mg/m^2^ (FEC) followed by 12 cycles of weekly paclitaxel 80 mg/m^2^ (wPTX). Sixteen of 102 patients showed HER2-positive breast cancer, and were administered weekly trastuzumab with wPTX. Patients underwent mastectomy or breast-conserving surgery after NAC. All patients who underwent breast-conserving surgery were administered postoperative radiotherapy. Overall survival time was set in days as the period from the initiation of NAC. DFS (disease-free survival) was defined as freedom from all local, regional, or distant recurrence. All patients were followed by physical examination, ultrasonography, computed tomography and bone scan. The median follow-up period was 6.2 months. This study was conducted with the approval of the ethical committee of Osaka City University, and written informed consent was obtained from all patients.

### Assessment of clinical and pathological responses to NAC

Clinical response of the primary tumor was assessed by ultrasonography, computed tomography, and physical examination after NAC. Clinical responses were classified according to the WHO criteria [34]. After NAC, patients underwent appropriate surgery. The clinical response to preoperative chemotherapy was determined from the two diameters measurable in two dimensions by multiplying the longest diameter by the greatest perpendicular diameter and was classified as follows. Clinical complete response (cCR) was judged as the disappearance of all known disease determined by two observations not less than four weeks apart. Clinical partial response (cPR) was a 50% or greater decrease in total tumor lesions. Clinical no change (cNC) was a less than 50% decrease in total tumor size, without a 25% increase in tumor size. Clinical progressive disease (cPD) was defined as a 25% or greater increase in the tumor size, or the appearance of new lesions. The first two categories, cCR and cPR, were judged as effective. Pathological responses of the tumor and dissected lymph nodes were classified according to the evaluation criteria of the Japanese Breast Cancer Society (JBCS) [35], using a 5 histological-grade scale (Grades 0, 1a, 1b, 2, and 3) as follows: Grade 0, no response or almost no change in cancer cells after treatment; Grade 1, slight response; Grade 1a, mild response, mild change in cancer cells regardless of the area, or marked changes in cancer cells in less than one-third of total cancer cells; Grade 1b, moderate response, marked changes in one-third or more but less than two-thirds of tumor cells; Grade 2, marked response or marked changes in two-thirds or more of tumor cells; and Grade 3, no residual tumor cells, necrosis or disappearance of all tumor cells, or replacement of all cancer cells by granuloma-like and/or fibrous tissue. pCR (pathological complete response) was defined as the complete disappearance of infiltrates, including lymph node infiltrates. Tumors with residual ductal carcinoma in situ were included in the pCR group. Marked changes approaching a complete response with only a few remaining cancer cells were classified as near pCR [36,37]. The others were classified in the non-pCR group.

### Immunohistochemical examinations

All patients underwent a CNB before NAC, and an operation consisting of mastectomy or conserving surgery with axillary lymph node dissection after NAC at Osaka City University. Tissues from each patient were fixed in buffered formalin and embedded in paraffin. Serial tissue sections of 4 μm thickness were stained with hematoxylin-eosin and used for immunohistochemical staining. Expressions of CA9, estrogen receptor (ER), progesterone receptor (PgR), and HER2 were assessed by immunohistochemistry. After the paraffin sections were deparaffinized, they were heated for 20 min at 105°C by autoclave in Target Retrieval Solution (Dako, Carpinteria, CA). After blocking with 10% goat serum, the slides were incubated with the primary monoclonal antibodies against each of CA9 (clone M75, 1:1000; Novus Biologicals), ER (clone 1D5, dilution 1:80; Dako, Cambridge, UK), PgR (clone PgR636, dilution 1:100; Dako), and HER2 (Hercep Test, Dako) overnight at 4 °C. Peroxidase was introduced using a streptavidin conjugate and then peroxidase reactivity was visualized using a DAB solution, followed by counterstaining with haematoxylin.

### Immunohistochemical assessment

**Figure 1:**
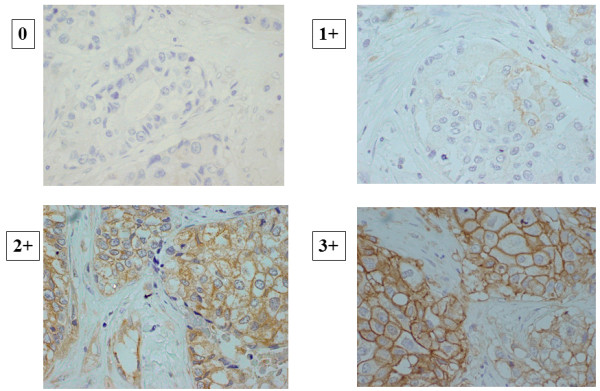
**Immunohistochemical determination of CA9 expression.** The positivity of a tumor for CA-9 was semi-quantitatively analyzed according to the percentage of cells showing membrane positivity. Score 0, negative staining in all cells; score 1+, weakly positive or focally positive staining in <10% of the cells; score 2+, moderately positive staining covering >10% of the cells; and score 3+, strongly positive staining, including >10% of the cells.

Immunohistochemical scoring was graded by trained pathologists (Masahiko Ohsawa and Kenichi Wakasa, Department of Diagnostic Pathology). The stroma was excluded from the staining evaluation. All staining was scored by counting the number of positive-stained cells, and was expressed as a percentage of the 1000 tumor cells counted across several representative fields of the section using a standard light microscope equipped with a × 100 square graticule. The reproducibility of counting was assessed by a second investigator. The cut-off for ER positivity and PgR positivity was ≥1% positive tumor cells with nuclear staining. HER2 was graded in four steps according to the accepted scheme: 0, 1+, 2+, 3+. HER2 was considered to be positive if immunostaining was 3+ or if a 2+ result showed gene amplification by fluorescent in situ hybridization. The ER, PR, and HER2 stainings were evaluated as described in previous reports [38]. The CA9 antibody intensely stained the membranes of cancer cells. Scores were applied as follows: score 0, negative staining in all cells; score 1+, weakly positive or focally positive staining in <10% of the cells; score 2+, moderately positive staining covering >10% of the cells; and score 3+, strongly positive staining in >10% of the cells (Figure 1). CA9 expression was considered positive for scores of 2+ or 3 + .

### Statistical analysis

Statistical analysis was performed using SPSS 13.0 statistical software (SPSS Inc., Chicago, IL). The association between the expression of CA9 and clinicopathological parameters was analyzed with the chi-square test. Binary logistic regression was used for multivariate analyses to identify independent prognostic factors for a pathological complete response. The Kaplan-Meier method was used to estimate the values of DFS. DFS was compared using a log-rank test. The Cox regression model was used for multivariate analysis of prognostic factors. In all of the tests, a *p* value less than 0.05 was considered to be statistically significant.

## Results

### Clinicopathological responses of breast cancers to NAC

**Figure 2:**
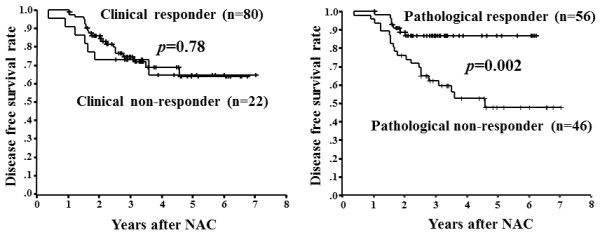
**Association between clinicopathologic response and disease-free survival.** Disease-free survival in pathologic non-responders was significantly (p = 0.002) shorter than that in pathologic responders, while the clinical response was not associated with disease-free survival (p = 0.78).

The cCR rate was 17% (18/102), cPR was 61% (62/102), cNC was 20% (20/102), and cPD was 2% (2/102). Therefore, the clinical responders (cCR + cPR) made up 78% (80/102) of the patients. The pathological response was evaluated using resected tissue after NAC. Of the tumors investigated, 12% (12/102) were histological response grade 1a, 33% (34/102) were grade 1b, 20% (20/102) were grade 2a, 16% (16/102) were grade 2b, and 20% (20/102) were grade 3. Patients were classified into pathologic responders (grade 2 and 3; 55% of all patients) and non-responders (grade 1; 45%) according to the grade of the tumor. The pCR rate was 29% (30/102). The DFS of pathologic non-responders was significantly (p = 0.01) shorter than that of pathologic responders, while no significant difference in DFS was found between clinical non-responders and clinical responders (Figure 2).

### Association between clinicopathological parameters and CA9 expression in CNB specimens

The CA9 expression of primary breast tumors before NAC was analyzed using CNB specimens. Of the 102 breast cancer patients, 47 patients (46%) had CA9-positive breast tumors, while 55 (54%) had CA9-negative tumors. Table 1 shows the correlation between clinicopathological parameters and CA9 expression in breast cancers. The CA9 expression in CNB specimens was significantly correlated with lymph node metastasis (70%, p = 0.001) and lymphatic invasion (69%, p = 0.003). The pCR rate of CA9-positive tumors (23%, 7/30) was significantly lower (p = 0.003) than that of CA9-negative tumors (77%, 23/30). The pathological non-responder tumors showed significantly more frequent CA9 expression than the pathological responder tumors (p < 0.001). Clinical response (cCR + cPR) was not associated with CA9 expression (p = 0.062). Recurrent tumors were observed in 28 of 102 patients. CA9 expression was significantly more frequent (p < 0.001) in patients with recurrent tumors (79%, 22/28) than in those with non-recurrent tumors (34%, 25/74). There was no significant association between CA9 expression and other clinicopathological factors.

**Table 1:** Correlations between CA9 expression and clinicopathological parameters in CNB of 102 primary breast cancers

		**CA9 expression**	
**Parameter**	**Positive**	**Negative**	
	**(n = 47)**	**(n = 55)**	***p*-value**
Age			
≥55	21 (42%)	29 (58%)	
<55	26 (50%)	26 (50%)	0.418
Menopause			
Positive	31 (44%)	40 (56%)	
Negative	16 (52%)	15 (48%)	0.459
Intrinsic subtype			
Luminal	22 (48%)	24 (52%)	
Luminal-HER	4 (50%)	4 (50%)	
HER2	4 (24%)	13 (76%)	0.209
Triple-negative	17 (55%)	14 (45%)	
Tumor size			
≥ 4 cm	12 (67%)	6 (33%)	
< 4 cm	35 (42%)	49 (58%)	0.053
Lymph node status			
Positive	23 (70%)	10 (30%)	
Negative	24 (35%)	45 (65%)	0.001
Lymph-vascular invasion			
Positive	20 (69%)	9 (31%)	
Negative	27 (37%)	46 (63%)	0.003
Nuclear grade (NG)			
NG1	32 (43%)	42 (57%)	
NG 2&3	15 (54%)	13 (46%)	0.350
Pathological response			
pCR	7 (23%)	23 (77%)	
Non-pCR	40 (56%)	32 (44%)	0.003
Responder (histological grade 2&3)	16 (29%)	40 (71%)	
non-Responder (histological grade 1)	31 (67%)	15 (33%)	<0.001
Clinical response			
Responder (cCR + cPR)	33 (41%)	47 (59%)	
non-Responder (cNC + cPD)	14 (64%)	8 (36%)	0.062
Recurrence			
Yes	22 (79%)	6 (21%)	
No	25 (34%)	49 (66%)	<0.001

### The correlation between the pCR and the pathological or clinical response

We examined the correlation between the pathological response (pCR vs non-pCR) and pathological or clinical response (Table 2). A pathological response was significantly (p < 0.001) associated with pCR, and a clinical response was also significantly (p = 0.018) associated with pCR.

**Table 2:** Correlations between the pathological and clinical response and the pCR

	**Pathological response**		
	**pCR**	**non-pCR**	***p*-value**
Pathological response			
Responder (histological grade 2&3)	30	26	
non-Responder (histological grade 1)	0	46	*p* < 0.001
Clinical response			
Responder (cCR + cPR)	28	52	
non-Responder (cNC + cPD)	2	20	*p* = 0.018

### Association between CA9 and pathological complete response in CNB specimens

Univariate analysis revealed that the expressions of CA9, ER, and PgR in CNB specimens were significantly associated with pCR. There was no significant association between pCR and the other clinicopathological factors. Multivariate analysis revealed that only CA9 expression was significantly associated with pCR (Table 3).

**Table 3:** Univariate and multivariate analyses of the pathological complete response in 102 breast cancers

**Parameter**		**Univariate analysis**			**Multivariate analysis**	
	**Odds ratio**	**95% CI**	***p *** **value**	**Odds ratio**	**95% CI**	***p *** **value**
CA9 expression in CNB						
positive vs negative	0.24	0.09-0.63	0.041	0.21	0.07-0.59	0.003
ER						
positive vs negative	0.24	0.10-0.61	0.002	0.23	0.02-2.37	0.219
PgR						
positive vs negative	0.22	0.08-0.61	0.004	0.36	0.10-1.30	0.120
HER2						
positive vs negative	1.75	0.68-4.48	0.244			
Molecular subtypes						
HR+/HER2- vs others	0.33	0.13-0.83	0.018	1.78	0.18-17.44	0.622
HR-/HER2+ vs others	0.02	0.01-2999894	0.728			
HR-/HER2+ vs others	1.75	0.683-4.483	0.244			
HR-/HER2- vs others	1.17	0.93-1.46	0.176			
Age						
≥55 vs <55	0.88	0.37-2.05	0.759			
Menopause						
positive vs negative	0.82	0.33-2.05	0.677			
Tumor size						
≥4 cm vs <4 cm	0.64	0.19-2.12	0.463			
Lymph node status						
positive vs negative	4.33	0.93-20.1	0.061			

### Correlation between clinicopathological parameters and disease-free survival

**Figure 3:**
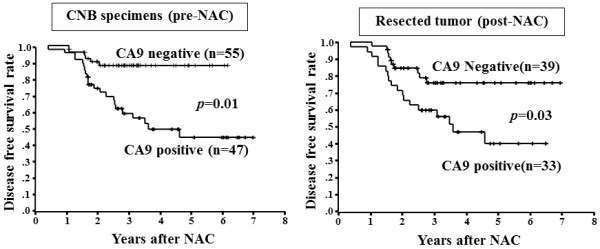
**Disease-free survival of patients based on CA-9 expression.** The Kaplan-Meier survival curve shows the disease-free survival in relation to the CA-9 expression. A statistically significant difference in the survival was observed between the CA-9-positive and CA-9-negative groups in both CNB specimens and resected tissues (log-rank, p = 0.01 and p = 0.03, respectively).

CA9 expression of breast tumors was analyzed using both CNB specimens and resected tissues. Since 30 of the 102 breast tumors showed a pathological complete response, these cases were excluded from the evaluation of CA9 expression. CA9 expression was therefore examined in 72 resected tissues after NAC. DFS in patients with CA9-positive tumors was significantly shorter than that in those with CA9-negative tumors in both samples (CNB specimens and resected tissues) (Figure 3). Univariate analysis revealed that CA9 expression in CNB specimens, CA9 expression in resected tissues, tumor size, lymph node status, and pathological response were significantly associated with DFS. There was no significant association between DFS and clinical response. Multivariate regression analyses indicated that CA9 expression in resected tissues after NAC was an independent prognostic factor for DFS (Table 4).

**Table 4:** Univariate and multivariate analysis of disease-free survival

**Parameter**		**Univariate analysis**			**Multivariate analysis**	
	**Odds ratio**	**95% CI**	***p *** **value**	**Odds ratio**	**95% CI**	***p *** **value**
CA9 expression in CNB specimens						
positive vs negative	4.44	1.80-10.9	0.001	2.32	0.88-6.08	0.089
CA9 expression in resected tissues						
positive vs negative	2.63	1.21-5.70	0.014	2.39	1.04-5.49	0.041
Tumor size						
≥ 4 cm vs < 4 cm	2.46	1.11-5.45	0.026	1.61	0.65-3.97	0.302
Lymph node status						
N1-3 vs N0	3.00	1.42-6.35	0.004	0.97	0.38-2.47	0.955
Lymph-vascular invasion						
positive vs negative	4.22	1.99-8.92	<0.001	2.75	1.11-6.62	0.028
ER						
positive vs negative	0.91	0.43-1.94	0.807			
PgR						
positive vs negative	0.95	0.45-2.02	0.905			
HER2						
positive vs negative	1.36	0.59-3.11	0.46			
Clinical response						
Responder vs non-responder	0.88	0.37-2.08	0.78			
Pathological response						
Responder vs non-responder	0.29	0.12-0.67	0.004	1.03	0.38-2.78	0.953
pCR vs non-pCR	0.19	0.45-0.81	0.024	1.34	0.27-6.62	0.722

## Discussion

In recent years, NAC has been adopted as one of the standard care regimens for primary resectable early-stage breast cancer. In such cases, the NAC generally consists of an anthracycline-containing regimen and taxane [4,5]. The evaluation of the tumor response to NAC is important to determine the appropriate post-operative chemotherapeutic regimen for patients with recurrent tumors. There are various systems for classifying the survival response and pathological response in neoadjuvant trials—i.e., the cTMN, Fisher’s, Chevailler’s, and JBCS systems—and all of these have been shown to yield basically comparable results [39]. In this study, we used the WHO and JBCS classifications as the therapeutic response criteria. The pCR rate was 29% (30/102), and the response rate was 78% (80/102). These response rates were similar to those previously reported [6,9,10].

The correlation between chemosensitivity and survival remains controversial. Some papers have reported that the prognostic factors included the clinical and pathological response to primary chemotherapy. On the other hand, at least one paper has reported that response classifications were inadequate as prognostic markers of the long-term outcome after NAC [39]. Our data indicated that clinical response was not a significant predictor of DFS. Although clinical examination provides approximate indicators of chemotherapy responses, histopathologic examination of specimens after chemotherapy is important to evaluate the accurate response or the prognosis [40-43]. A tumor diagnosed as showing a complete clinical response sometimes retains residual carcinoma cells by microscopic examination; conversely, a palpable residual mass may show fibrosis without cancer cells [42,43]. These findings might explain why the association between clinical response and DFS was more less statistically significant than that between pathological response and DFS in our study.

Our data indicated that pathological response was a ignificant predictor of the DFS. In this study, FEC followed by wPTX was the only NAC regimen used for patients with resectable early-stage breast cancer. However, the variety of chemotherapy regimens used as NAC in previous reports might have been a factor in producing these inconsistent results.

CA9, a hypoxia-associated endogenous protein, has been implicated in the regulation of the hypoxic microenvironment [44,45]. CA9 is considered to be one of the cellular biomarkers of hypoxic regions in solid tumors. In the present analysis, CA9 was positive in CNB specimens from 47 (46%) of 102 patients, similar to the ratio in a previous study [46]. CA9 expression was significantly associated with lymph node status and lymph-vascular invasion. CA9 has been shown to maintain the survival of breast tumor cells under hypoxic conditions [47]. Breast cancer cells under hypoxic conditions might be associated with aggressive tumor phenotypes, which may indicate a poor prognosis for patients with CA9-positive breast cancer, as suggested in previous studies [29,30,48-50].

Tan et al. reported that CA9 in basal-like breast tumors was associated with resistance to chemotherapy (cyclophosphamide, methotrexate and 5-fluorouracil (CMF) or adriamycin and cyclophosphamide (AC)) and poor prognosis [30]. In our present analysis of 31 triple-negative breast cancers, the DFS of patients with CA9-positive tumors was significantly shorter (p = 0.015) than that of patients with CA9-negative tumors (Additional file 2: Figure S1). The chemosensitivity of triple-negative breast cancer patients with CA9 was significantly lower than that of the CA9-negative cases (Additional file 1: Table S2). CA9 might be a useful biomarker for chemotherapy in triple-negative breast cancer. Supuran and colleagues found that selective CA9 inhibitors inhibited cell migration and spreading of breast cancer cells in the absence of oxygen, suggesting that CA9 is a pivotal target for antitumor therapy in patients with breast carcinoma [25,51]. These findings suggest that CA9 inhibitors followed by wPTX chemotherapy might be useful in cases of breast carcinoma with resistance to FEC.

Biological markers predicting chemosensitivity have been evaluated in several studies, but there is still no clinically useful marker. ER- or PR-positive patients showed lower pCR rates after NAC than ER- or PR-negative patients. The pCR rate of CA9-positive tumors in CNB specimens was significantly lower than that of CA9-negative tumors. Multivariate analysis revealed that CA9 expression before NAC was an independent predictive factor for pCR. An extensive hypoxic microenvironment as determined by CA9 expression in breast cancer might play a significant role in the resistance to chemotherapy. These results may indicate that CA9 expression in CNB specimens is a useful marker for predicting chemosensitivity to NAC.

We also examined the correlation of CA9 expression between CNB tissues and resected tissues in the 72 patients. Although no significant correlation of CA9 staining was observed between the two groups, CA9 expression in resected tissues showed a tendency (p = 0.081) toward association with that in CNB tissues (Additional file 1: Table S3). CA9 expression after NAC (67%) was higher than that before NAC (46%). CA9-positive cells were observed more frequently in tumor specimens than in CNB specimens. Eleven of 32 patients with CA9-negative tumors before NAC were found to have CA9-positive tumors after NAC. NAC was thus effective in reducing CA9-negative cells, and resulted in an increase in hypoxic CA9-positive cells. Twenty-three of 55 patients with CA9-negative tumors before NAC achieved pCR, and could not be enrolled in the CA9 expression analysis because there was no tumor involvement detected in the resected tissues. These changes in CA9 expression before and after NAC might be one of the reasons for the lack of a significant correlation between the CA9 expression in CNB tissues and that in resected tissues.

CA9 expression in resected tissues after NAC was correlated with both prognosis and recurrence. In addition, multivariate regression analyses indicated that the CA9 expression level after NAC was an independent prognostic factor for DFS. Thus, CA9 expression after NAC may be a clinically informative prognostic marker for breast cancer patients treated with NAC. On the other hand, CA9 expression before NAC in CNB specimens may be a useful surrogate marker for predicting chemosensitivity. Our results indicate that the hypoxic marker CA9 in CNB specimens could be used to predict chemosensitivity, and that high expression of CA9 in resected tissue is correlated with worse outcomes in patients treated with FEC followed by wPTX chemotherapy.

## Conclusion

Hypoxic microenvironment as determined by CA9 expression in breast cancer might play a significant role in the resistance to chemotherapy, which indicates that CA9 expression in CNB specimens is a useful marker for predicting chemosensitivity to NAC. CA9 expression in resected tissue is prognostic of DFS in patients with resectable early-stage breast cancer treated by sequential FEC and weekly paclitaxel prior to resection.

## Abbreviations

CR: Complete response; CNB: Core needle biopsy; CSCs: Cancer stem cells; DFS: Disease-free survival; ER: Estrogen receptor; FEC: 5-fluorouracil + epirubicin + cyclophosphamide; HER2: Human epidermal growth factor receptor 2; IHC: Immunohistochemistry; NAC: Neoadjuvant chemotherapy; NC: No change; OS: Overall survival; PgR: Progesterone receptor; PR: Partial response; PD: Progressive disease; pCR: Pathologic complete response.

## Competing interests

The authors declare that they have no competing interests.

## Authors’ contributions

MY: study design, data analysis, paper preparation. NA: performance of experiments, data analysis, paper preparation. SK, HK, and TT: material sampling. MO, KW: pathological diagnosis. KH: manuscript review. All authors read and approved the final manuscript.

## Pre-publication history

The pre-publication history for this paper can be accessed here:

http://www.biomedcentral.com/1471-2407/14/400/prepub

## Supplementary Material

Additional file 1: Table S1 Clinicopathologic features of 102 breast cancers. **Table S2.** Correlations between CA9 expression and chemosensitivity in 31 triple-negative breast cancers. **Table S3.** Correlation of CA9 expression before NAC to that after NAC in the 72 patients who did not achieve pCR. Click here for file

Additional file 2: Figure S1 Disease-free survival of patients based on CA-9 expression in 31 cases of triple-negative breast cancer. Among the cases of triple-negative breast cancer, the DFS of patients with CA9-positive tumors was significantly shorter (p = 0.015) than that of patients with CA9-negative tumors. (TIFF 25 kb) Click here for file

## References

[B1] Mayer EL Carey LA Burstein HJ Clinical trial update: implications and management of residual disease after neoadjuvant therapy for breast cancer Breast Cancer Res 2007 9 5 110 10.1186/bcr1755 17888189 PMC2242653

[B2] Sachelarie I Grossbard ML Chadha M Feldman S Ghesani M Blum RH Primary systemic therapy of breast cancer Oncologist 2006 11 6 574 589 10.1634/theoncologist.11-6-574 16794237

[B3] Fisher B Brown A Mamounas E Wieand S Robidoux A Margolese RG Cruz AB Jr Fisher ER Wickerham DL Wolmark N DeCillis A Hoehn JL Lees AW Dimitrov NV Effect of preoperative chemotherapy on local-regional disease in women with operable breast cancer: findings from national surgical adjuvant breast and bowel project B-18 J Clin Oncol 1997 15 7 2483 2493 9215816 10.1200/JCO.1997.15.7.2483

[B4] Wolmark N Wang J Mamounas E Bryant J Fisher B Preoperative chemotherapy in patients with operable breast cancer: nine-year results from national surgical adjuvant breast and bowel project B-18 J Natl Cancer Inst Monogr 2001 30 96 102 11773300 10.1093/oxfordjournals.jncimonographs.a003469

[B5] Bear HD Anderson S Brown A Smith R Mamounas EP Fisher B Margolese R Theoret H Soran A Wickerham DL Wolmark N The effect on tumor response of adding sequential preoperative docetaxel to preoperative doxorubicin and cyclophosphamide: preliminary results from national surgical adjuvant breast and bowel project protocol B-27 J Clin Oncol 2003 21 22 4165 4174 10.1200/JCO.2003.12.005 14559892

[B6] Smith IC Heys SD Hutcheon AW Miller ID Payne S Gilbert FJ Ah-See AK Eremin O Walker LG Sarkar TK Eggleton SP Ogston KN Neoadjuvant chemotherapy in breast cancer: significantly enhanced response with docetaxel J Clin Oncol 2002 20 6 1456 1466 10.1200/JCO.20.6.1456 11896092

[B7] Goldhirsch A Wood WC Gelber RD Coates AS Thurlimann B Senn HJ Progress and promise: highlights of the international expert consensus on the primary therapy of early breast cancer 2007 Ann Oncol 2007 18 7 1133 1144 10.1093/annonc/mdm271 17675394

[B8] Fisher CS Ma CX Gillanders WE Aft RL Eberlein TJ Gao F Margenthaler JA Neoadjuvant chemotherapy is associated with improved survival compared with adjuvant chemotherapy in patients with triple-negative breast cancer only after complete pathologic response Ann Surg Oncol 2012 19 1 253 258 10.1245/s10434-011-1877-y 21725686 PMC3892697

[B9] Chollet P Amat S Cure H de Latour M Le Bouedec G Mouret-Reynier MA Ferriere JP Achard JL Dauplat J Penault-Llorca F Prognostic significance of a complete pathological response after induction chemotherapy in operable breast cancer Br J Cancer 2002 86 7 1041 1046 10.1038/sj.bjc.6600210 11953845 PMC2364175

[B10] Jones RL Smith IE Neoadjuvant treatment for early-stage breast cancer: opportunities to assess tumour response Lancet Oncol 2006 7 10 869 874 10.1016/S1470-2045(06)70906-8 17012049

[B11] Evans TR Yellowlees A Foster E Earl H Cameron DA Hutcheon AW Coleman RE Perren T Gallagher CJ Quigley M Crown J Jones AL Highley M Leonard RC Mansi JL Phase III randomized trial of doxorubicin and docetaxel versus doxorubicin and cyclophosphamide as primary medical therapy in women with breast cancer: an anglo-celtic cooperative oncology group study J Clin Oncol 2005 23 13 2988 2995 10.1200/JCO.2005.06.156 15860854

[B12] Bear HD Anderson S Smith RE Geyer CE Jr Mamounas EP Fisher B Brown AM Robidoux A Margolese R Kahlenberg MS Paik S Soran A Wickerham DL Wolmark N Sequential preoperative or postoperative docetaxel added to preoperative doxorubicin plus cyclophosphamide for operable breast cancer: national surgical adjuvant breast and bowel project protocol B-27 J Clin Oncol 2006 24 13 2019 2027 10.1200/JCO.2005.04.1665 16606972

[B13] Estevez LG Cuevas JM Anton A Florian J Lopez-Vega JM Velasco A Lobo F Herrero A Fortes J Weekly docetaxel as neoadjuvant chemotherapy for stage II and III breast cancer: efficacy and correlation with biological markers in a phase II, multicenter study Clin Cancer Res 2003 9 2 686 692 12576436

[B14] Chuthapisith S Eremin JM El-Sheemy M Eremin O Neoadjuvant chemotherapy in women with large and locally advanced breast cancer: chemoresistance and prediction of response to drug therapy Surgeon 2006 4 4 211 219 10.1016/S1479-666X(06)80062-4 16892838

[B15] Ross JS Symmans WF Pusztai L Hortobagyi GN Breast cancer biomarkers Adv Clin Chem 2005 40 99 125 16355921 10.1016/s0065-2423(05)40003-7

[B16] Colleoni M Viale G Zahrieh D Pruneri G Gentilini O Veronesi P Gelber RD Curigliano G Torrisi R Luini A Intra M Galimberti V Renne G Nole F Peruzzotti G Goldhirsch A Chemotherapy is more effective in patients with breast cancer not expressing steroid hormone receptors: a study of preoperative treatment Clin Cancer Res 2004 10 19 6622 6628 10.1158/1078-0432.CCR-04-0380 15475452

[B17] Rody A Karn T Gatje R Ahr A Solbach C Kourtis K Munnes M Loibl S Kissler S Ruckhaberle E Holtrich U von Minckwitz G Kaufmann M Gene expression profiling of breast cancer patients treated with docetaxel, doxorubicin, and cyclophosphamide within the GEPARTRIO trial: HER-2, but not topoisomerase II alpha and microtubule-associated protein tau, is highly predictive of tumor response Breast 2007 16 1 86 93 10.1016/j.breast.2006.06.008 17010609

[B18] Liao SY Brewer C Zavada J Pastorek J Pastorekova S Manetta A Berman ML DiSaia PJ Stanbridge EJ Identification of the MN antigen as a diagnostic biomarker of cervical intraepithelial squamous and glandular neoplasia and cervical carcinomas The American journal of pathology 1994 145 3 598 609 8080042 PMC1890321

[B19] Pastorek J Pastorekova S Callebaut I Mornon JP Zelnik V Opavsky R Zat’ovicova M Liao S Portetelle D Stanbridge EJ Cloning and characterization of MN, a human tumor-associated protein with a domain homologous to carbonic anhydrase and a putative helix-loop-helix DNA binding segment Oncogene 1994 9 10 2877 2888 8084592

[B20] Opavsky R Pastorekova S Zelnik V Gibadulinova A Stanbridge EJ Zavada J Kettmann R Pastorek J Human MN/CA9 gene, a novel member of the carbonic anhydrase family: structure and exon to protein domain relationships Genomics 1996 33 3 480 487 10.1006/geno.1996.0223 8661007

[B21] Chen CL Chu JS Su WC Huang SC Lee WY Hypoxia and metabolic phenotypes during breast carcinogenesis: expression of HIF-1alpha, GLUT1, and CAIX Virchows Archiv: an international journal of pathology 2010 457 1 53 61 10.1007/s00428-010-0938-0 20526721

[B22] Shin KH Diaz-Gonzalez JA Russell J Chen Q Burgman P Li XF Ling CC Detecting changes in tumor hypoxia with carbonic anhydrase IX and pimonidazole Cancer biology & therapy 2007 6 1 70 75 17172824 10.4161/cbt.6.1.3550

[B23] Russell J Carlin S Burke SA Wen B Yang KM Ling CC Immunohistochemical detection of changes in tumor hypoxia Int J Radiat Oncol Biol Phys 2009 73 4 1177 1186 10.1016/j.ijrobp.2008.12.004 19251089 PMC2680715

[B24] Ivanov S Liao SY Ivanova A Danilkovitch-Miagkova A Tarasova N Weirich G Merrill MJ Proescholdt MA Oldfield EH Lee J Zavada J Waheed A Sly W Lerman MI Stanbridge EJ Expression of hypoxia-inducible cell-surface transmembrane carbonic anhydrases in human cancer The American journal of pathology 2001 158 3 905 919 10.1016/S0002-9440(10)64038-2 11238039 PMC1850340

[B25] Neri D Supuran CT Interfering with pH regulation in tumours as a therapeutic strategy Nat Rev Drug Discov 2011 10 10 767 777 10.1038/nrd3554 21921921

[B26] Svastova E Hulikova A Rafajova M Zat'ovicova M Gibadulinova A Casini A Cecchi A Scozzafava A Supuran CT Pastorek J Pastorekova S Hypoxia activates the capacity of tumor-associated carbonic anhydrase IX to acidify extracellular pH FEBS Lett 2004 577 3 439 445 10.1016/j.febslet.2004.10.043 15556624

[B27] Swietach P Hulikova A Vaughan-Jones RD Harris AL New insights into the physiological role of carbonic anhydrase IX in tumour pH regulation Oncogene 2010 29 50 6509 6521 10.1038/onc.2010.455 20890298

[B28] Grabmaier K AdW MC Verhaegh GW Schalken JA Oosterwijk E Strict regulation of CAIX(G250/MN) by HIF-1alpha in clear cell renal cell carcinoma Oncogene 2004 23 33 5624 5631 10.1038/sj.onc.1207764 15184875

[B29] Betof AS Rabbani ZN Hardee ME Kim SJ Broadwater G Bentley RC Snyder SA Vujaskovic Z Oosterwijk E Harris LN Horton JK Dewhirst MW Blackwell KL Carbonic anhydrase IX is a predictive marker of doxorubicin resistance in early-stage breast cancer independent of HER2 and TOP2A amplification Br J Cancer 2012 106 5 916 922 10.1038/bjc.2012.32 22333602 PMC3305967

[B30] Tan EY Yan M Campo L Han C Takano E Turley H Candiloro I Pezzella F Gatter KC Millar EK O'Toole SA McNeil CM Crea P Segara D Sutherland RL Harris AL Fox SB The key hypoxia regulated gene CAIX is upregulated in basal-like breast tumours and is associated with resistance to chemotherapy Br J Cancer 2009 100 2 405 411 10.1038/sj.bjc.6604844 19165203 PMC2634728

[B31] Curran S Murray GI Matrix metalloproteinases: molecular aspects of their roles in tumour invasion and metastasis Eur J Cancer 2000 36 13 Spec No 1621 1630 10959048 10.1016/s0959-8049(00)00156-8

[B32] Graeber TG Osmanian C Jacks T Housman DE Koch CJ Lowe SW Giaccia AJ Hypoxia-mediated selection of cells with diminished apoptotic potential in solid tumours Nature 1996 379 6560 88 91 10.1038/379088a0 8538748

[B33] Singletary SE Greene FL Revision of breast cancer staging: the 6th edition of the TNM Classification Semin Surg Oncol 2003 21 1 53 59 10.1002/ssu.10021 12923916

[B34] Miller AB Hoogstraten B Staquet M Winkler A Reporting results of cancer treatment Cancer 1981 47 1 207 214 10.1002/1097-0142(19810101)47:1<207::AID-CNCR2820470134>3.0.CO;2-6 7459811

[B35] Kurosumi M Akashi-Tanaka S Akiyama F Komoike Y Mukai H Nakamura S Tsuda H Histopathological criteria for assessment of therapeutic response in breast cancer (2007 version) Breast Cancer 2008 15 1 5 7 10.1007/s12282-007-0016-x 18224386

[B36] Toi M Nakamura S Kuroi K Iwata H Ohno S Masuda N Kusama M Yamazaki K Hisamatsu K Sato Y Kashiwaba M Kaise H Kurosumi M Tsuda H Akiyama F Ohashi Y Takatsuka Y Phase II study of preoperative sequential FEC and docetaxel predicts of pathological response and disease free survival Breast Cancer Res Treat 2008 110 3 531 539 10.1007/s10549-007-9744-z 17879158

[B37] Nishimura R Osako T Okumura Y Hayashi M Arima N Clinical significance of Ki-67 in neoadjuvant chemotherapy for primary breast cancer as a predictor for chemosensitivity and for prognosis Breast Cancer 2010 17 4 269 275 10.1007/s12282-009-0161-5 19730975

[B38] Aomatsu N Yashiro M Kashiwagi S Takashima T Ishikawa T Ohsawa M Wakasa K Hirakawa K CD133 is a useful surrogate marker for predicting chemosensitivity to neoadjuvant chemotherapy in breast cancer PloS one 2012 7 9 e45865 10.1371/journal.pone.0045865 23049880 PMC3457956

[B39] Shien T Shimizu C Seki K Shibata T Hojo T Ando M Kohno T Katsumata N Akashi-Tanaka S Kinoshita T Fujiwara Y Comparison among different classification systems regarding the pathological response of preoperative chemotherapy in relation to the long-term outcome Breast Cancer Res Treat 2009 113 2 307 313 10.1007/s10549-008-9935-2 18286370

[B40] Pinder SE Provenzano E Earl H Ellis IO Laboratory handling and histology reporting of breast specimens from patients who have received neoadjuvant chemotherapy Histopathology 2007 50 4 409 417 10.1111/j.1365-2559.2006.02419.x 17448015

[B41] Sahoo S Lester SC Pathology of breast carcinomas after neoadjuvant chemotherapy: an overview with recommendations on specimen processing and reporting Archives of pathology & laboratory medicine 2009 133 4 633 642 19391665 10.5858/133.4.633

[B42] Fisher ER Wang J Bryant J Fisher B Mamounas E Wolmark N Pathobiology of preoperative chemotherapy: findings from the national surgical adjuvant breast and bowel (NSABP) protocol B-18 Cancer 2002 95 4 681 695 10.1002/cncr.10741 12209710

[B43] Feldman LD Hortobagyi GN Buzdar AU Ames FC Blumenschein GR Pathological assessment of response to induction chemotherapy in breast cancer Cancer Res 1986 46 5 2578 2581 3697997

[B44] Saarnio J Parkkila S Parkkila AK Haukipuro K Pastorekova S Pastorek J Kairaluoma MI Karttunen TJ Immunohistochemical study of colorectal tumors for expression of a novel transmembrane carbonic anhydrase, MN/CA IX, with potential value as a marker of cell proliferation The American journal of pathology 1998 153 1 279 285 10.1016/S0002-9440(10)65569-1 9665489 PMC1852958

[B45] Helmlinger G Sckell A Dellian M Forbes NS Jain RK Acid production in glycolysis-impaired tumors provides new insights into tumor metabolism Clin Cancer Res 2002 8 4 1284 1291 11948144

[B46] Bartosova M Parkkila S Pohlodek K Karttunen TJ Galbavy S Mucha V Harris AL Pastorek J Pastorekova S Expression of carbonic anhydrase IX in breast is associated with malignant tissues and is related to overexpression of c-erbB2 J Pathol 2002 197 3 314 321 10.1002/path.1120 12115877

[B47] Potter CP Harris AL Diagnostic, prognostic and therapeutic implications of carbonic anhydrases in cancer Br J Cancer 2003 89 1 2 7 10.1038/sj.bjc.6600936 12838292 PMC2394207

[B48] Span PN Bussink J Manders P Beex LV Sweep CG Carbonic anhydrase-9 expression levels and prognosis in human breast cancer: association with treatment outcome Br J Cancer 2003 89 2 271 276 10.1038/sj.bjc.6601122 12865916 PMC2394253

[B49] Chia SK Wykoff CC Watson PH Han C Leek RD Pastorek J Gatter KC Ratcliffe P Harris AL Prognostic significance of a novel hypoxia-regulated marker, carbonic anhydrase IX, in invasive breast carcinoma J Clin Oncol 2001 19 16 3660 3668 11504747 10.1200/JCO.2001.19.16.3660

[B50] Generali D Fox SB Berruti A Brizzi MP Campo L Bonardi S Wigfield SM Bruzzi P Bersiga A Allevi G Milani M Aguggini S Dogliotti L Bottini A Harris AL Role of carbonic anhydrase IX expression in prediction of the efficacy and outcome of primary epirubicin/tamoxifen therapy for breast cancer Endocr Relat Cancer 2006 13 3 921 930 10.1677/erc.1.01216 16954440

[B51] Gieling RG Babur M Mamnani L Burrows N Telfer BA Carta F Winum JY Scozzafava A Supuran CT Williams KJ Antimetastatic effect of sulfamate carbonic anhydrase IX inhibitors in breast carcinoma xenografts J Med Chem 2012 55 11 5591 5600 10.1021/jm300529u 22621623

