# Supplementary material for: Carbonic anhydrase 9 is associated with chemosensitivity and prognosis in breast cancer patients treated with taxane and anthracycline
Source: BMC Cancer. 2014 Jun 4;14:400. doi: 10.1186/1471-2407-14-400 (PMC4058694; doi:10.1186/1471-2407-14-400)
Supplement: Additional file 1: Table S1 — Clinicopathologic features of 102 breast cancers. Table S2. Correlations between CA9 expression and chemosensitivity in 31 triple-negative breast cancers. Table S3. Correlation of CA9 expression before NAC to that after NAC in the 72 patients who did not achieve pCR. [file 1471-2407-14-400-S1.docx]

**Supplement Table 1.** Clinicopathologic features of 102 breast cancers

| Parameter | (n=102) | (%) |
| --- | --- | --- |
| Age |  |  |
| ≥55 | 50 | (49) |
| <55 | 52 | (51) |
| Menopause |  |  |
| Positive | 71 | (69) |
| Negative | 31 | (31) |
| Intrinsic subtype |  |  |
| Luminal | 46 | (45) |
| Luminal-HER | 8 | (8) |
| HER2 | 17 | (17) |
| Triple-negative | 31 | (30) |
| Tumor size |  |  |
| ≥ 4 cm | 18 | (18) |
| < 4 cm | 84 | (82) |
| Lymph node status |  |  |
| Positive | 33 | (32) |
| Negative | 69 | (68) |

**Supplement Table 2.** Correlations between CA9 expression and chemosensitivity in 31 triple-negative breast cancers

|  |  | All breast cancers (n=102) | | | TNBC (n=31) | | | non-TNBC (n=71) | | |
| --- | --- | --- | --- | --- | --- | --- | --- | --- | --- | --- |
|  |  | CA9 | |  | CA9 | |  | CA9 | |  |
| Parameter | vs | positive | negative | *p* value | positive | negative | *p* value | positive | negative | *p* value |
|  |  | (n=47) | (n=55) |  | (n=17) | (n=14) |  | (n=30) | (n=41) |  |
| Age | ≧55 | 21 | 29 | 0.418 | 9 | 9 | 0.524 | 12 | 20 | 0.463 |
|  | ＜55 | 26 | 26 |  | 8 | 5 |  | 18 | 21 |  |
|  |  |  |  |  |  |  |  |  |  |  |
| Menopause | positive | 31 | 40 | 0.459 | 12 | 12 | 0.316 | 19 | 28 | 0.663 |
|  | negative | 16 | 15 |  | 5 | 2 |  | 11 | 13 |  |
|  |  |  |  |  |  |  |  |  |  |  |
| Tumor size | ≧4cm | 12 | 6 | 0.053 | 3 | 1 | 0.607 | 9 | 5 | 0.063 |
|  | ＜4cm | 35 | 49 |  | 14 | 13 |  | 21 | 36 |  |
|  |  |  |  |  |  |  |  |  |  |  |
| Lymph node status | Positive | 23 | 10 | 0.001 | 8 | 1 | 0.021 | 15 | 9 | 0.014 |
|  | Negative | 24 | 45 |  | 9 | 13 |  | 15 | 32 |  |
|  |  |  |  |  |  |  |  |  |  |  |
| Lymph-vascular invasion | positive | 20 | 9 | 0.003 | 9 | 1 | 0.007 | 11 | 8 | 0.107 |
|  | Negative | 27 | 46 |  | 8 | 13 |  | 19 | 33 |  |
|  |  |  |  |  |  |  |  |  |  |  |
| Nuclear grade (NG) | NG1 | 32 | 42 | 0.350 | 6 | 12 | 0.009 | 26 | 30 | 0.169 |
|  | NG2&3 | 15 | 13 |  | 11 | 2 |  | 4 | 11 |  |

**Supplement Table 3.** Correlation of CA9 expression before NAC to that after NAC in the 72 patients who did not achieve pCR.

|  |  | CA9 expression in resected tissues | |  |
| --- | --- | --- | --- | --- |
|  |  | Negative | Positive | *p*-value |
| CA9 expression in CNB specimens | Negative | 21 | 11 |  |
|  | Positive | 18 | 22 | *p* = 0.081 |
