# Supplementary figures and images for: Carbonic anhydrase 9 is associated with chemosensitivity and prognosis in breast cancer patients treated with taxane and anthracycline
Source: BMC Cancer. 2014 Jun 4;14:400. doi: 10.1186/1471-2407-14-400 (PMC4058694; doi:10.1186/1471-2407-14-400)

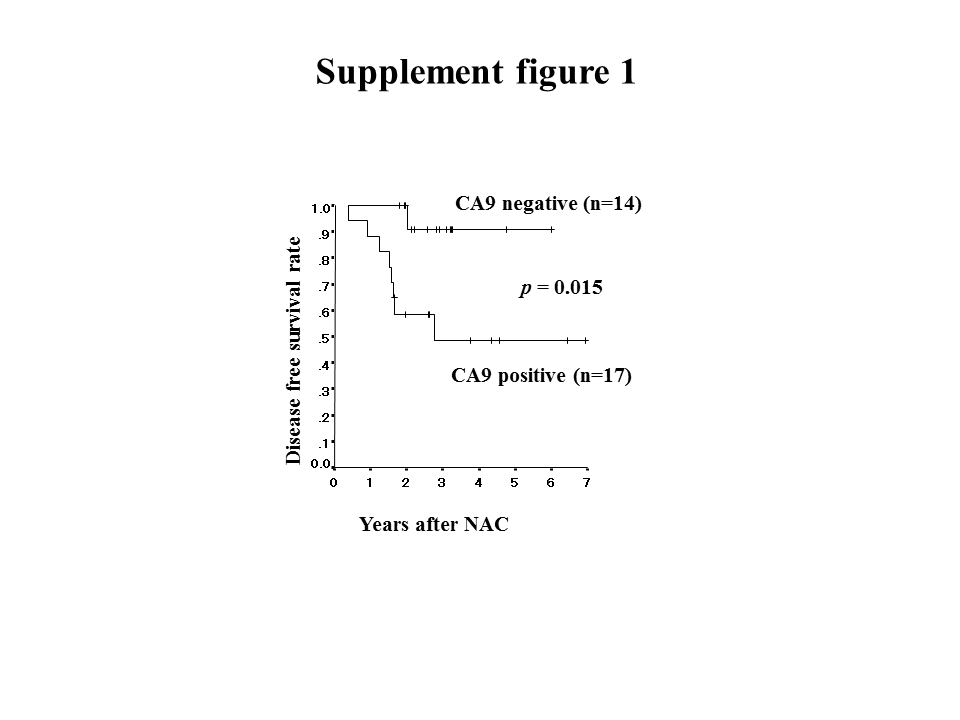

Supplement: Additional file 2: Figure S1 — Disease-free survival of patients based on CA-9 expression in 31 cases of triple-negative breast cancer. Among the cases of triple-negative breast cancer, the DFS of patients with CA9-positive tumors was significantly shorter (p = 0.015) than that of patients with CA9-negative tumors. (TIFF 25 kb) [file 1471-2407-14-400-S2.tiff]
